# Antioxidant and Neuroprotective Effects of Carnosine: Therapeutic Implications in Neurodegenerative Diseases

**DOI:** 10.3390/antiox11050848

**Published:** 2022-04-26

**Authors:** Cristina Solana-Manrique, Francisco José Sanz, Guillermo Martínez-Carrión, Nuria Paricio

**Affiliations:** 1 Departamento de Genética, Facultad CC Biológicas, Universidad de Valencia, 46100 Burjassot, Spain; crisoman@alumni.uv.es (C.S.-M.); fco.sanz@uv.es (F.J.S.); guimarc2@alumni.uv.es (G.M.-C.); 2 Instituto Universitario de Biotecnología y Biomedicina (BIOTECMED), Universidad de Valencia, 46100 Burjassot, Spain

**Keywords:** carnosine, neuroprotective effect, oxidative stress, protein aggregation, inflammation, neurodegenerative diseases, Parkinson’s disease, Alzheimer’s disease, aging, therapeutic approach

## Abstract

Neurodegenerative diseases (NDs) constitute a global challenge to human health and an important social and economic burden worldwide, mainly due to their growing prevalence in an aging population and to their associated disabilities. Despite their differences at the clinical level, NDs share fundamental pathological mechanisms such as abnormal protein deposition, intracellular Ca^2+^ overload, mitochondrial dysfunction, redox homeostasis imbalance and neuroinflammation. Although important progress is being made in deciphering the mechanisms underlying NDs, the availability of effective therapies is still scarce. Carnosine is a natural endogenous molecule that has been extensively studied during the last years due to its promising beneficial effects for human health. It presents multimodal mechanisms of action, being able to exert antioxidant, anti-inflammatory and anti-aggregate activities, among others. Interestingly, most NDs exhibit oxidative and nitrosative stress, protein aggregation and inflammation as molecular hallmarks. In this review, we discuss the neuroprotective functions of carnosine and its implications as a therapeutic strategy in different NDs. We summarize the existing works that study alterations in carnosine metabolism in Alzheimer’s disease and Parkinson’s disease, the two most common NDs. In addition, we review the beneficial effect that carnosine supplementation presents in models of such diseases as well as in aging-related neurodegeneration.

## 1. Introduction

Neurodegenerative diseases (NDs) constitute a global challenge to human health and important social and economic burdens worldwide, mainly due to their growing prevalence in an aging population and to their associated disabilities. Clinically, NDs are characterized by the progressive damage and death of nerve cells from a particular region of the brain, resulting in disease-specific clinical symptoms. They are devastating disorders that lead to severe impairments of patients’ abilities such as cognitive decline, dementia or motor deficits, and ultimately to death [1]. Despite their differences at the clinical level, NDs share fundamental pathological mechanisms such as abnormal protein deposition, intracellular Ca^2+^ overload, mitochondrial dysfunction, redox homeostasis imbalance and neuroinflammation, among others [2,3]. Although important progress is being made in deciphering the mechanisms underlying NDs, the availability of effective therapies is still scarce. Indeed, current therapies generally lack disease-modifying activity and act to alleviate symptoms, but they cannot delay or halt the progress of the disease. Therefore, exploring new therapeutic options is urgently required to identify new treatments for NDs either using synthetic or natural products or even existing drugs. Obviously, the existence of shared pathological mechanisms leading to neurodegeneration in different NDs reinforces the importance of those pathways as common targets for intervention strategies [4]. Therefore, therapeutic approaches directed to reduce oxidative stress (OS) levels, protein aggregation or neuroinflammation could be beneficial for diverse NDs [1].

Carnosine is a natural endogenous molecule discovered more than 100 years ago as an abundant non-protein, nitrogen-containing compound of meat [5]. However, it has been extensively studied during the last years due to its promising beneficial effects for human health [6,7]. Carnosine (β-alanyl-L-histidine) is a histidine-containing dipeptide (HCD) that, together with its analogs homocarnosine, anserine and ophidine/balenine, is widely distributed in mammalian tissues [8,9]. This dipeptide is mainly located in skeletal and cardiac muscles but is also present in the brain. Interestingly, all these tissues exhibit a very active oxidative metabolism. However, while most studies have been directed to determine the role of carnosine in muscles, its physiological role in the brain is still unclear (reviewed in [7,9,10]). Carnosine is present throughout the brain but is particularly enriched in the olfactory bulb and cortex. β-alanine (β-ala) and L-histidine (L-his) are the two amino acids forming carnosine, which can be easily obtained from the circulatory system for its synthesis and have different origins. While β-ala is synthesized in the liver or can be obtained from the diet in humans, L-His must be ingested since it cannot be synthesized de novo [9]. In addition, they can be obtained from proteolysis of endogenous proteins [11]. Both can be delivered to the brain via the circulatory system and cross the blood–brain barrier (BBB) through amino acid transporters for carnosine synthesis. For example, β-ala transport in the brain seems to occur via the β-amino acid transporter [12]. Carnosine synthesis takes place specifically in glial cells, mostly in oligodendrocytes; however, neurons and astrocytes are the main utilizers of this dipeptide in the brain [10]. In addition, it is assumed that the majority of brain carnosine comes from de novo synthesis, despite the fact that it can also cross BBB and can penetrate this tissue when it comes from the diet [7,10].

Growing evidence coming from in vivo and in vitro studies has indicated the protective role of carnosine on human diseases and aging due to its multimodal mechanisms of action that involve several pathways (see Section 3). Indeed, carnosine is especially effective in reducing OS and inflammation and inhibiting protein carbonylation, glycosylation and aggregation [9,13]. Consequently, it has been proposed that this dipeptide could be tested in the treatment of diverse diseases such as NDs, cancer, diabetes, schizophrenia and especially those characterized by elevated OS levels and inflammation [8,9,10,14,15,16]. Indeed, the favorable effects of carnosine have been demonstrated in in vivo and in vitro models of human diseases as well as in patients after direct supplementation with this dipeptide [7,9,10,13,17]. Interestingly, they were also confirmed in genetic models with mutations in genes involved in endogenous carnosine metabolism [18,19]. Taken together, these observations confirm the therapeutic potential of carnosine for several chronic diseases, including NDs.

In this review, we discuss the neuroprotective functions of carnosine and its implications as a therapeutic strategy in different NDs. For conducting this, we first focus on carnosine metabolism, on its biochemical properties and describe the multifaceted physiological roles of this dipeptide, especially in the brain. Subsequently, we address the pathophysiological relevance of alterations in carnosine metabolism in human diseases, mainly in NDs. Finally, we discuss the potential of targeting carnosine metabolism and of carnosine itself as a promising therapeutic approach for the most prevalent NDs and aging-related neurodegeneration.

## 2. Proteins Involved in Carnosine Metabolism and Transport

In this section, we describe all proteins that participate in carnosine homeostasis. They are not only enzymes involved in its synthesis, modification and degradation, but they are also proteins responsible for transmembrane transport of carnosine (Table 1). In humans, these processes require the activity of several proteins: one for its synthesis, two for its degradation, one for its methylation and at least five transporters [9,20,21].

**Figure 1:**
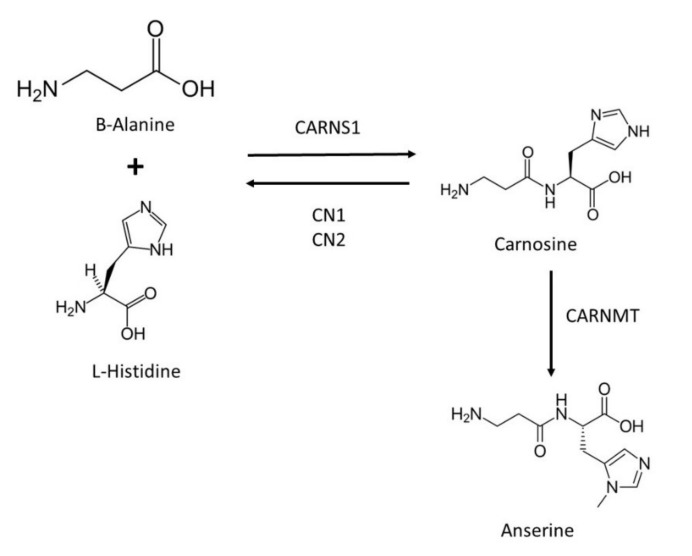
Schematic illustration of carnosine metabolism.

The synthesis of carnosine starting from its constituent amino acids, β-ala and L-his depends on the activity of carnosine synthase 1 (CARNS1; EC 6.3.2.11), at the cost of adenosine 5′-triphosphate (ATP) hydrolysis (Figure 1). This enzyme belongs to the ATP-grasp superfamily of adenosine 5′-diphosphate (ADP)-producing ligases [22], and it is also called ATPGD1 (from ATP-Grasp domain containing protein 1). CARNS1 is a cytosolic enzyme present in skeletal and cardiac muscles and the olfactory bulb in the brain [22]. As mentioned above, carnosine is produced in glial cells, being the only oligodendrocyte brain cells that express *CARNS1* and present CARNS1 activity in humans (reviewed in [9,20]). Regarding degradation, carnosine and other HCDs are hydrolyzed by specific enzymes called carnosinases (CN), also known as cytosolic nonspecific dipeptidases (CNDP) (Figure 1). In humans, two dipeptidase isoforms belonging to the M20 metalloprotease family are able to degrade carnosine: serum carnosinase (CN1 or CNDP1; EC 3.4.13.20), and tissue carnosinase (CN2 or CNDP2; EC 3.4.13.18). CN1 occurs in human serum and has a narrow substrate spectrum for HCDs, since it specifically degrades carnosine and its analogs anserine and homocarnosine and is mainly expressed in the liver but also in the brain, for which its activity is concentrated in oligodendrocytes. CN2 is a Mn^2+^ dependent cytosolic enzyme located intracellularly, which has broader substrate specificity than CN1 and presents ubiquitous expression in human tissues but at very low levels in the brain [20,23]. In addition, carnosine can be also transformed into its analog anserine through the carnosine N-methyltransferase enzyme (CARNMT; EC 2.1.1.22) [24] (Figure 1). This enzyme also methylates other HCDs such as homocarnosine and is weakly expressed in brain cells (reviewed in [9]).

Carnosine can be transported across cellular membranes by a number of proteins from the proton-coupled oligopeptide transporter (POT) family, also called solute carrier family 15 (SLC15) [20]. In humans, several transmembrane proteins of the SLC15 family can transport di- and tripeptides including carnosine such as SLC15A1 (also known as peptide transporter 1, PEPT1), SLC15A2 (also known as peptide transporter 2, PEPT2), SLC15A4 (also known as peptide/histidine transporter 1, PHT1) and SLC15A3 (also known as peptide/histidine transporter 2, PHT2) [9,20]. All these proteins can transport carnosine, which is responsible for either its uptake or its release [25]. Expression data corresponding to these transporters and carnosine-related enzymes described above and obtained from RNA sequencing analyses of human and mouse brain cells [26,27] are available at http://www.brainrnaseq.org (accessed on 2 March 2022). A detailed analysis of the expression of those genes in brain cells is included in [9]. This analysis showed that the expression levels of genes encoding carnosine transporters in humans are quite low in general; the detectable expression of *SLC15A2* and *SLC15A4* is found in microglia, while mature astrocytes only express *SLC15A2* [9]. Neurons, in comparison, express these transporters at very low levels but maybe enough to import carnosine. Indeed, it has been recently demonstrated that the speed of active transport of carnosine into neurons through SLC15A2 is ten-times greater than that of passive transport, thus indicating that these cells have a sufficient amount of the transporter for carnosine uptake [28]. A recent study has demonstrated that carnosine can be also transported by SLC22A15 [21], a sodium-dependent transporter of the SLC22A family highly expressed in oligodendrocytes compared with other brain cells [27]. Other transporters such as SLC15A1 can transport dietary carnosine into the apical side of intestinal cells, where it can be hydrolyzed by CN2 to produce L-his and β-ala or it can remain unchanged and enter the blood [11]. Brain expression data of genes encoding all these proteins and of protein themselves have been obtained from The Human Protein Atlas webpage (https://www.proteinatlas.org/, accessed on 2 March 2022) [29,30] (Table 1).

Pointing out the relevance of carnosine in the brain, the expression and/or activity of proteins involved in its metabolism were shown to be altered in several NDs and in aging (see Section 4). For example, CN2 was shown to be overexpressed in the substantia nigra pars compacta (SNpc) of brains of Parkinson’s disease (PD) patients [31]. Similarly, CN1 expression, and activity increased in murine models of aging [8]. In both cases, a decrease in carnosine levels will reduce its beneficial and neuroprotective effects. Therefore, strategies based on extending the half-life of endogenous carnosine through CN-activity inhibitors or on designing carnosine-like molecules that retain its beneficial biological actions, but being resistant with respect to CN degradation, could have therapeutic potential [32].

## 3. Mechanism of Action of Carnosine

**Figure 2:**
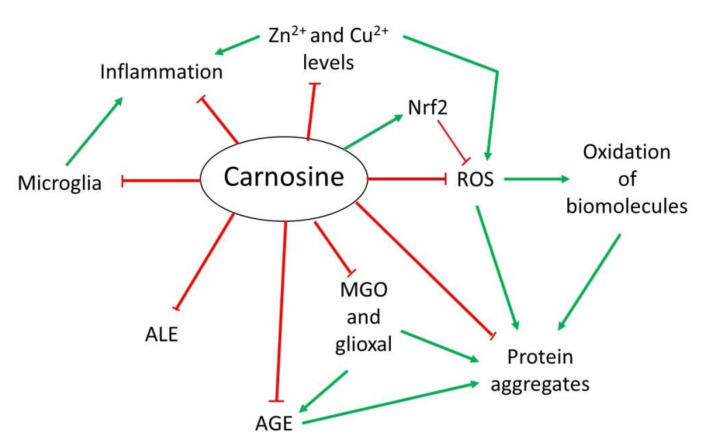
Overview of the mechanism of action of carnosine as antioxidant, anti-aggregant, anti-glycation and anti-inflammatory molecule.

Over the years, several properties have been assigned to carnosine. However, although it is a multifunctional dipeptide, it is mainly described to play an important role as a molecule with antioxidant, anti-aggregant and anti-inflammatory activities [9,13]. Here, we discuss the most interesting mechanisms of action of carnosine, especially those associated with the nervous system that are relevant in the context of NDs (Figure 2).

### 3.1. Antioxidant Activity

OS is defined as an imbalance between cellular production of reactive oxygen species (ROS) and the mechanisms that remove these species [33]. Principal detoxification pathways include antioxidant tripeptide glutathione (GSH) and the activity of the superoxide dismutase 1 (SOD1) enzyme, among others [33,34]. However, an important antioxidant role has been also assigned to carnosine in the last years [9,13]. Due to its composition, this dipeptide presents in its structure an imidazole ring that belongs to the this residue, responsible for its activity as a ROS scavenger and its protective capacity against hypochlorous acid (HOCl) [9,13,20]. Indeed, it has been demonstrated that carnosine is oxidized to 2-oxo-carnosine in SH-SY5Y human neuroblastoma cells stably expressing CARNS1 after H_2_O_2_ exposure through the oxidation of the imidazole ring. A similar modification was detected for its analogs homocarnosine and anserine [35]. Furthermore, 2-oxo-carnosine seems to have a stronger antioxidant effect than GSH, one of the main cellular detoxifying mechanisms [34,35]. Carnosine can also protect cells against HOCl, another toxic species produced from H_2_O_2_ and Cl^−^ in mammalian cells, mainly in leukocytes. In excess, HOCl is toxic to cells since it can modify proteins. In fact, it has been linked to several disorders such as Alzheimer’s disease (AD) and cancer associated with inflammation [36]. The imidazole ring of carnosine rapidly reacts with HOCl producing an imidazole chloramine, thus limiting its oxidative activity in a highly efficient manner [13,20,37].

However, in addition to its direct antioxidant mechanism, recent studies have demonstrated that carnosine may indirectly exert its antioxidant function through different molecular pathways. Carnosine can modulate the activity of Nrf2 (nuclear factor erytroid 2-related factor 2), the main regulator of the cellular antioxidant response [13,38,39,40]. In OS conditions, carnosine is able to increase Nrf2 expression and translocation to the nucleus, where it regulates the transcription of hundreds of genes that contain an antioxidant response element (ARE) in their promoter region, such as thioredoxin 1, SOD1 or catalase [13,41]. Currently, it is unclear how carnosine produces Nrf2 pathway activation [13]. Nevertheless, a recent study performed in podocytes from a mouse model of diabetes mellitus demonstrated that carnosine also activates the PI3K (phosphatidylinositol 3-kinase)/AKT (protein kinase B) pathway, which can result in Nrf2 activation [38].

### 3.2. Anti-Glycating and Anti-Aggregant Activity

OS significantly contributes to the impairment of neuronal cells function through modifications of biomolecules, such as oxidation of lipids, DNA or proteins. Among them, proteins are the main targets of ROS due to the presence of residues that can easily react with these species, producing the addition of carbonyl groups [42]. In addition to proteins, the carbonylation of sugars and lipids leads to the formation of advanced glycation end-products (AGEs) and advanced lipoxidation end-products (ALEs), respectively, which are a cluster of heterogeneous molecules generated in a non-enzymatic reaction that play an important role in several NDs [13,20,43]. Carnosine can inhibit AGEs and ALEs formation by detoxifying reactive carbonyl species through its imidazole ring, in a similar manner than ROS [13,43]. In this context, carnosine has been proved useful in preventing the reactivity of methylglyoxal (MGO) and glyoxal, which are carbonyl compounds that can produce protein glycation and aggregation [13,44]. In particular, carnosine was shown to reduce OS and carbonylation levels in rats treated with MGO [45]. As for antioxidant activity, the increase in Nrf2 pathway activity by carnosine should also be considered in the carbonyl quenching mechanism. Nrf2 activates the expression of several antioxidant enzymes and oxidoreductases, which convert carbonyl compounds in less reactive products such as alcohols or carboxyl derivatives [13]. Interestingly, the activation of Nrf2 also explains the ability of carnosine to decrease AGEs formation from MGO and glyoxal [43].

In addition to the effect of MGO regarding AGEs and ALEs, this compound can also produce protein aggregation and deposition leading to neuron death in some NDs [20]. There is evidence that carnosine prevents oxidation and glycation, both of which contribute to the crosslinking of proteins. Excitingly, a direct anti-crosslinking activity has been attributed to carnosine that also depends on the imidazole ring [20,44,46]. In addition, carnosine has been shown to exhibit protease activity in cell extracts from nervous and cardiac cell from rats [20]. In sum, this dipeptide could present therapeutic potential for NDs in which protein aggregation plays an important physiopathological role.

### 3.3. Anti-Inflammatory and Metal Ion Chelator Activity

Some NDs are characterized by neuroinflammation produced by microglial cells and astrocytes that, along with the formation of protein aggregates, ultimately leads to cell death [47,48]. In this respect, a possible anti-inflammatory activity of carnosine through the regulation of pro-inflammatory cytokine release was described in human epithelial CaCo-2 cells [49]. Although it is still unknown how carnosine produced this effect, it could be due to its ability to form complexes with metal ions, particularly with zinc (Zn^2+^) [20]. Zn is an essential mineral that is required as a cofactor of several enzymes, in cell proliferation and in wound healing [50]. Carnosine can form a complex with Zn^2+^ called polaprezinc, a molecule that exhibits several beneficial properties, such as anti-inflammatory effect in the colon through inhibition of NF-κB (nuclear factor kappa-light-chain-enhancer of activated B cells) signaling, reduction in interleukin-8 release, and induction in Hsp72 [50,51,52]. Currently, polaprezinc is widely used for Zn supplementation therapy and to protect the mucosa against ulceration. Therefore, due to the link between Zn and NDs and considering the neuroprotective effects of carnosine, this complex has been proposed to be used in NDs to reduce inflammation through its chelator activity [51]. Regarding the brain, it has also been reported that carnosine may regulate Zn and copper (Cu) homeostasis in the synaptic cleft. The dipositive ions of these metals are necessary to modulate synaptic transmissions; for example, Zn^2+^ and Cu^2+^ can inhibit NMDA (N-methyl-D-aspartate)-type glutamate receptors in the post-synaptic membrane. It has been shown that carnosine directly binds to Zn^2+^ and Cu^2+^, thus regulating the synaptic impulse [20,51].

## 4. Alterations of Carnosine Homeostasis in Neurodegenerative Diseases

It is considered that aging is one of the most important risk factors to develop NDs [53]. Aging is characterized by an increase in OS levels and in inflammatory response [54]. Since carnosine has been reported to exert protective effects addressing both alterations, it might play an important role in aging. In fact, increased CN1 expression levels and activity were found in a mouse model of aging [55]. Given the tight relation between aging and NDs, it is likely that these diseases also present disturbances in carnosine metabolism. In this Section, we present results obtained in different studies indicating that alterations in carnosine homeostasis can be associated with the onset and/or progression of NDs.

### 4.1. Alzheimer’s Disease

Alzheimer’s disease (AD) is the most common ND worldwide. It is characterized by progressive cognitive decline, which worsens patients’ quality of life and affects activities of their daily living [56,57]. It is considered that AD presents a multifactorial etiology. However, the formation of extracellular amyloid-beta (Aβ) peptide aggregates (commonly referred to as the senile plaques) as well as intracellular tau-related neurofibrillary tangles might play important roles in the development of the disease [58,59]. In addition, it has been shown that Aβ aggregates are able to alter Ca^2+^ homeostasis, to increase OS levels and are related with the inflammatory response [56]. Moreover, metabolic alterations have been linked to AD pathogenesis since AGEs were also found in Aβ plaques [60].

To date, very few studies have been carried out to evaluate carnosine homeostasis in AD, although there is growing evidence that its metabolism could be relevant in such disease [10]. Several years ago, a study was performed to evaluate the level of free amino acids and dipeptides in probable AD (pAD) patients compared with control subjects [61]. The results showed that carnosine and anserine levels were lower and higher, respectively, in plasma from pAD patients. However, when quantified together the total amount of carnosine, anserine was reduced in pAD patients. In addition, they showed that levels of carnosine and anserine precursors were lower in cerebrospinal fluid (CSF) and serum from pAD patients than in those from control subjects. It was suggested that these alterations could be due to a reduction in CARNS1 activity or to an enhancement of CN activity [61]. In contrast, a proteomic assay performed in CSF from AD patients and control subjects demonstrated that CN1 levels were reduced in patients [62]. This result was further confirmed by an ELISA (enzyme-linked immunoassay) test; as a result, reduced CN1 levels were proposed as a biomarker of early AD [62]. Moreover, Balion et al.(2007) studied whether CN activity was altered in AD patients and people suffering mixed dementia (MD) [63]. No significant differences in CN activity were found between AD or MD patients and control subjects. However, patients suffering MD presented reduced CN activity compared with AD patients. Therefore, it was proposed that CN activity could be useful to differentiate AD from MD [63]. In a different study, the levels of the β-ala amino acid, one of carnosine’s constituents, were evaluated in serum from elderly people (between 60 and 79 years old). Results showed that individuals who developed AD or vascular dementia presented a reduction in β-ala levels. In such scenarios, it was suggested that reduced β-ala levels could be related with a reduced intake of carnosine, hence supporting a neuroprotective role for this dipeptide [64]. Additionally, animal models have been also used to study carnosine metabolism. It has been recently found that carnosine levels were reduced in the brains of young PLB4 female mice, a rodent model of AD [65].

Overall, studies performed so far indicate that there is a reduction in carnosine levels in AD patients along with a reduction in CN activity. Therefore, low carnosine levels could be due to a reduction in its synthesis via CARNS1. In addition, results reported in the above-mentioned studies suggest that carnosine could exert beneficial effects in AD. However, further studies will be required to fully understand carnosine metabolism alterations in AD.

### 4.2. Parkinson’s Disease

Parkinson’s disease (PD) is the second most common ND [66]. It is characterized by the appearance of motor symptoms such as resting tremor, bradykinesia and rigidity [67]. However, non-motor symptoms such as sleep disturbances and cognitive defects are also observed in PD patients, worsening their quality of life [67,68]. PD is caused by the selective loss of dopaminergic neurons in SNpc and by a reduction in dopamine in the striatum [69]. In addition, the formation of intracellular α-synuclein protein aggregates (known as Lewy bodies) in the surviving neurons is also characteristic of this disease [70]. The mechanisms that lead to neurodegeneration in PD are still unclear. However, mitochondrial alterations, increased OS levels, endoplasmic reticulum stress, excitotoxicity, neuroinflammation, formation of protein aggregates and metabolic alterations might play an important role in the development of the disease [71,72,73].

Even though carnosine properties and activities can affect directly most processes involved in PD onset/development, the metabolism of this dipeptide has been barely studied in PD models and patients. For example, Wassif et al. (1994) analyzed CN1 activity in serum of PD patients, finding that it was reduced compared with that of control individuals [74]. In consequence, this might lead to carnosine accumulation in PD patients. Conversely, a proteomic assay performed by Licker et al. (2012) showed that CN2 was overexpressed in the SNpc of PD patients, which was subsequently confirmed by Western blot and immunohistochemical analyses [31]. In addition, they also studied if CN2 levels in CSF could be a reliable biomarker for PD. However, no differences in CN2 levels were observed in PD patients compared with control subjects [31]. In addition, several studies carried out in animal models of PD also suggested the existence of alterations in carnosine metabolism. As an example, a metabolomic study recently performed in our laboratory in a *Drosophila* PD model showed an increase in β-ala levels and a reduction in his levels in model flies compared to the controls [75]. In such a scenario, our hypothesis is that increased β-ala levels could be due to an increase in carnosine degradation, which may lead to a reduction in carnosine levels and its neuroprotective effect. In addition, we propose that these levels might be reduced by increased activity/expression of histidine decarboxylase, an enzyme that converts his into the neurotransmitter histamine [76]. Histamine is a neurotransmitter that has a role in the modulation of innate immune response, leading to microglial migration and cytokine release [77]. Indeed, the overactivation of microglial cells leads to neuronal damage observed in PD [78]. Further experiments are being performed in our laboratory to confirm our hypotheses, which will validate the existence of alterations in carnosine metabolism in PD model flies (unpublished results). Supporting this assumption, an increase in histamine levels was found in post-mortem brain samples of patients with PD [79].

Increased levels of CNDP2 specifically in the SNpc suggest that carnosine metabolism might play a key role in PD. In addition, alterations in the levels of β-ala and his, the carnosine precursors, found in a *Drosophila* PD model support that carnosine levels might be altered. Therefore, these results reinforce the necessity to continue investigating this molecule to elucidate its relevance in PD onset/progression.

## 5. Therapeutic Potential of Carnosine in Neurodegenerative Diseases

Although carnosine is mainly located in muscle tissues, the presence of this molecule and other HCDs in the brain has arisen as a promising research subject due to the beneficial properties of those molecules. As mentioned in Section 3, carnosine exhibits antioxidant, anti-aggregant and anti-inflammatory activities; therefore, it could be beneficial for NDs since most of them present OS and nitrosative stress, protein aggregation and inflammation as molecular hallmarks [9]. Although the above-mentioned properties have been widely demonstrated and reviewed [9,10], there is no consensus on the role that carnosine homeostasis could play in the etiology of NDs. In this section, we will focus on reviewing the use of carnosine supplements on several models of NDs and aging-associated neurodegeneration and the therapeutic potential that this molecule may bear for the most common neurodegenerative processes (Table 2). In addition, we will also mention studies carried out in humans in which carnosine supplementation was beneficial in the context of NDs.

### 5.1. Alzheimer’s Disease

Several groups have used either in vitro or in vivo models of AD in order to determine the effect of carnosine on some molecular features of this disease. Regarding in vitro models, Preston et al. (1998) showed that carnosine protected endothelial cells against Aβ toxicity [86]. It was also demonstrated that it prevented the formation of amyloid fibrils and decreased the number of Aβ aggregates by interacting with the hydrophobic core of that peptide [80,81]. A novel approach consisting of conjugating carnosine with other molecules such as hyaluronic acid has also been shown to be quite effective in avoiding the formation of Aβ aggregates [82]. In addition, carnosine was able to reduce OS levels induced by oligomeric Aβ by decreasing intracellular NO and O₂^−^ levels and the expression of enzymes such as iNOS in BV-2 microglial cells. It also improved the inflammatory state of those cells by decreasing the secretion of pro-inflammatory cytokines and increasing the production of TGF-β (transforming growth factor β), an anti-inflammatory cytokine [85]. It has been also proved that carnosine is able to induce the production of neurotrophins such as NGF (nerve growth factor) and BDNF (brain-derived neurotrophic factor) in glial cells [83] and can avoid degradation by inhibiting metalloproteases that process such neurotrophins [84]. Accordingly, several studies have pointed to the relationship between impairments or deficits of neurotrophins and AD development [102,103].

As indicated above, the effects of carnosine supplementation have also been evaluated in vivo using animal models of AD. One of them was developed in *Caenorhabditis elegans*, in which Aβ was overexpressed in large muscle cells. Carnosine inhibited Aβ aggregation in AD model worms by triggering a cytosolic unfolded protein response through HSF-1 transcription factors and downstream chaperones. In doing so, carnosine improved protein homeostasis and suppressed AD-related phenotypes in those worms [87]. In addition, carnosine is virtually harmless in mice and humans also tolerate it well [9]. Supplementation with this molecule in several rodent models of AD has shown improvements in some of the molecular alterations associated with the disease. As an example, Corona et al. (2011) were able to observe a reduction in Aβ accumulation in the central nervous system of a triple transgenic mice model of AD after carnosine supplementation [88]. That effect was accompanied by a decrease in lipid peroxidation, a rescue of mitochondrial function and an improvement of the cognitive deficits in those animals [88]. Later, a different study on streptozotocin-induced diabetic rats demonstrated a reduction in acetylcholinesterase activity in hippocampal neurons and an impairment of NF-kB pro-inflammatory and pro-oxidative signaling after carnosine supplementation, supporting the antioxidant and anti-inflammatory effects of this dipeptide in order to treat NDs [89]. Another group administered carnosine to a transgenic mice model of AD fed with high fat diet. They found that it was able to restore the RAGE (AGE receptor) expression levels, to suppress microglia activation and to prevent AD-derived cognitive decline [90]. More recently, Hegazy et al. (2022) compared the effect of carnosine and exercise in rat AD model generated by intracerebroventricular injection of streptozotocin [91]. The results obtained in this study correlated with the previously reported beneficial effects of carnosine. Indeed, carnosine administration was able to reduce soluble Aβ concentration and tau phosphorylation, as well as to improve insulin and BDNF signaling [91].

### 5.2. Parkinson’s Disease

Other studies have assessed the potential of carnosine to treat PD. Next, we present some of the results obtained in these studies using cell and animal models of PD. Zhao et al. (2017) used a salsolinol-induced cell model of PD developed in endothelial rat brain cells, and they were able to observe a protective effect of carnosine administration [92]. Specifically, they found a decrease in apoptosis and mitochondria-derived ROS levels, together with a recovery of lipid peroxidation and levels of antioxidant enzymes [92]. More recently, a group working with GT1-7 cells, an immortalized cell line of hypothalamic neurons, examined cell death induction by 6-hydroxydopamine (6-OHDA), which is mediated through ROS production. They evaluated whether carnosine could prevent such cell death, finding that, due to its antioxidant properties, carnosine was able to reduce ROS generation through JNK (Jun N-terminal kinase) pathway inhibition as well as to suppress the stress response and release of pro-inflammatory cytokines. All these effects resulted in an increased survival of carnosine-treated cells [93]. A different study, in which rats were injected with 6-OHDA in the striatum, showed similar results. Carnosine pre-treatment increased the antioxidant ability of the cells by upregulating enzymes such as catalase and decreased malondialdehyde and nitrites levels to a physiological-like state, thus resulting in an the attenuation of generalized apoptosis [94]. The beneficial effects of carnosine have also been demonstrated in animal models of PD. For example, carnosine pre-intake was shown to reduce pro-oxidative and pro-inflammatory markers in a 1-methyl-4-phenyl-1,2,3,6-tetrahydropyridine (MPTP)-treated mice. The specific effects observed included the upregulation of antioxidant enzymes, such as SOD and glutathione peroxidase (GPX), and a reduction in inflammatory cytokines [95]. Carnosine supplementation was also tested in a genetic mice model of PD based on α-synuclein overexpression (Thy1-aSyn) [96]. In this study, the attenuation of mitochondrial and ribosomal defects was observed after intranasal carnosine treatment due to a transcriptional upregulation of mitochondrial proteins belonging to complexes I, IV and V [96]. This model of intranasal administration has been proved to be more effective to treat NDs than other models that are supplemented with oral carnosine because intranasal delivery avoids serum CN1 function and bypasses the blood–brain barrier [96]. The anti-aggregate properties of carnosine were subsequently demonstrated in the same mice model of PD. It was confirmed that intranasal carnosine administration was able to slow significant motor deficits progression and α-synuclein accumulation [97]. Brown et al. (2021) recently obtained similar conclusions using the same model [98]. They could demonstrate that intranasal-administered carnosine was able to mitigate motor deficits in a dose-dependent manner in PD model mice by decreasing α-synuclein accumulation in the SNpc [98]. Regarding the anti-inflammatory effect of carnosine, a recent study demonstrated that the intranasal administration of a complex formed with carnosine and α-lipoic acid induced neuroprotection in PD model rats after MPTP treatment [104]. This complex restored the levels of intermediate products from dopamine and serotonine metabolism, thus constituting a novel therapeutic strategy for this disease [104]. On the other hand, carnosine supplementation has been also evaluated in PD patients treated with L-DOPA therapy [105]. It was found that patients supplemented with 1.5 g per day of carnosine for 30 days showed a reduction in neurological symptoms and of OS markers levels in blood samples [105]. These results supported the beneficial effects of carnosine as a complement to DOPA drug therapy in PD patients.

### 5.3. Aging-Related Neurodegeneration

Aging is an unavoidable fate for almost every complex living being. Furthermore, in the context of the occidental lifestyle with medical access and technological advances where lifespan increased drastically, it has become one of the main threats to our wellbeing [53]. One of the most sensitive cells to aging includes neurons, in which this process shares most of the pathological pathways known to be involved in NDs [6]. Elevated OS levels derived from metabolism is characteristic of aging; this can lead to impaired glial cells function and result in neuroinflammation, thus leading to aging-associated neuronal loss [106]. Carnosine, due to its antioxidant and anti-inflammatory properties, has gained relevance as a probable anti-aging molecule that could be useful to slow down aging-related neurodegeneration.

It has been shown that the endogenous levels of carnosine decrease during aging in brain [107]. Therefore, this group thought of supplementing aged Wistar rats with carnosine in order to check if it could alleviate age-related phenotypes. The results obtained in this study indicated that carnosine was able to attenuate aging-induced Aβ plaque formation and improve cognitive impairment in elderly rats [101]. In addition, galactose treatment has been stablished as a model of accelerated aging in rats since it leads to an increase in OS levels. Carnosine supplementation was able to decrease galactose-induced OS in male rats [100]. Similar results were obtained in an in vitro model of accelerated aging also induced with galactose. This study showed that neuronal cells differentiated with retinoic acid from SH-SY5Y cells treated with carnosine and L-his, one of its precursors, were able to upregulate antioxidant enzymes such as GPX and SOD and to reduce cleaved caspase 3, pro-inflammatory cytokines mRNA levels and Aβ peptide expression [99]. The effect of carnosine supplementation has also been evaluated in elderly subjects. For instance, it has been demonstrated that an increase in carnosine and anserine intake improved the cognitive function and physical capacity of subjects older than 65 years compared to non-supplemented individuals [108]. Similarly, a supplementation of 1 g per day of carnosine/anserine in 60–78-year-old subjects preserved verbal episodic memory function (considered a sensitive test for detecting a cognitive decline) and reduced the levels of inflammatory cytokines in blood [109].

## 6. Conclusions

Carnosine is an endogenous dipeptide that has been widely studied due to its multiple effects. Among them, carnosine has been shown to exert antioxidant, anti-inflammatory, anti-glycating and anti-aggregant properties [9,13,20]. Interestingly, most of these activities directly impact processes such as OS, neuroinflammation, protein glycation/aggregation, etc., which are relevant for the onset/development of several NDs [2,3]. However, carnosine metabolism has been scarcely studied in such diseases. Despite this, the results obtained to date point to a general reduction in carnosine levels in NDs such as AD or PD (see Section 4). Interestingly, alterations in carnosine metabolism have been also related to other disorders such as diabetes or cardiac alterations [110,111]. Several approaches have been carried out to increase carnosine levels in models of those diseases. For instance, it has been reported that CARNS1 overexpression in mice was able to protect heart from ischemia reperfusion injury [18]. In addition, compounds able to reduce CN activity such as bestatin, an allosteric CN2 inhibitor, have been reported to be beneficial in the retina of a diabetes mouse model [112].

Currently, existing therapies for NDs are only symptomatic and are not able to reduce or stop their progression [4]. Given the multimodal mechanism of action of carnosine, along with the finding that its levels are reduced in several NDs, this dipeptide has been postulated as a promising therapeutic for such diseases. In fact, carnosine supplementation has been evaluated in several models of AD and PD, in humans, as well as in aging-related neurodegeneration, finding that it was able to alleviate some of their most characteristic phenotypes (see Section 5 and Table 2). In addition, one clinical trial was conducted to evaluate carnosine supplementation in three patients with multiple sclerosis (NCT03995810). Results showed that, at eight weeks of supplementation, it was able to attenuate symptomatology in those individuals [113]. Another clinical trial was initiated in 2017 in early-stage PD patients (NCT03330470); however, no results have been published yet. Therefore, the therapeutic potential of alternative strategies already used in other human diseases and directed toward increasing endogenous carnosine levels should be further evaluated in NDs such as PD and AD.

## Figures and Tables

**Table 1:** Human proteins involved in carnosine metabolism.

Name	Symbols	Function	Predicted Location	Brain Expression ^a^
Carnosine synthase 1	CARNS1, ATPGD1, KIAA1394	Synthesis	Intracellular	RNA: oligodendrocytes Protein: subsets of glial cells, possibly oligodendrocytes
Carnosine dipeptidase 1 (or serum carnosinase)	CN1, CNDP1, CPGL2, HsT2308, MGC10825	Degradation	Intracellular, secreted	RNA: oligodendrocytes Protein: subsets of glial and neuronal cells
Carnosine dipeptidase 2 (or tissue carnosinase)	CN2, CNDP2, CPGL, FLJ10830,H2T2298, PEPA	Degradation	Intracellular	RNA: very low levels Protein: no data available
Carnosine N-methyltransferase 1	CARNMT1, C9orf41, FLJ25795	Methylation	Intracellular	RNA: very low levels Protein: no data available
Solute carrier family 15 member 1	SLC15A1, PEPT1, HPEPT1, HEPCT1	Transport	Membrane	RNA: no expression Protein: no data available
Solute carrier family 15 member 2	SLC15A2, PEPT2	Transport	Membrane	RNA: astrocytes, microglia Protein: no data available
Solute carrier family 15 member 3	SLC15A3, hPTR3, PHT2	Transport	Membrane	RNA: very low levels Protein: no data available
Solute carrier family 15 member 4	SLC15A4, PTR4, PHT1	Transport	Membrane	RNA: oligodendrocytes, microglia and several neurons Protein: no data available
Solute carrier family 22 member 15	SLCA22A15, FLIPT1	Transport	Membrane	RNA: oligodendrocytes and low in astrocytes Protein: no data available

Data obtained from The Human Protein Atlas website (https://www.proteinatlas.org/, accessed on 28 February 2022). ^a^ Information extracted from “RNA single cell type specificity” and “Human brain protein location” sections.

**Table 2:** Beneficial effects of carnosine supplementation in models of Alzheimer’s disease, Parkinson’s disease and aging.

Alzheimer’s Disease			
Type of model	Description	Phenotypes modified	References
Cellular models	Cells supplemented with Aβ peptide or endogenously overexpressing Aβ	Reduction in Aβ aggregation, inflammation and OS, and neurotrophins induction	[80,81,82,83,84,85,86]
*C. elegans*	Aβ overexpression in large muscle cells	Induction of cytosolic unfolded proteins response	[87]
Rodents	Transgenic AD model mice Streptozotocin-induced AD rats	Reduction in cognitive impairment, OS, pro-inflammatory signaling, microglia activation and Aβ accumulation	[88,89,90,91]
**Parkinson Disease**			
**Type of model**	**Description**	**Phenotypes modified**	**References**
Cellular model	Salsolinol-treated rat brain endothelial cells GT1-7 hypothalamic immortalized neurons treated with 6-OHDA	Increase in survival and upregulation of antioxidant enzymes Reduction in apoptosis, ROS levels, lipid peroxidation and pro-inflammatory cytokines	[92,93]
Rodents	Mice and rats treated with MPTP or 6-OHDA Mice model overexpressing α-synuclein (Thy1-aSyn)	Increase in antioxidant enzymes, improvement of mitochondrial function Reduction in α-synuclein aggregation, motor deficits and apoptosis	[94,95,96,97,98]
**Aging**			
**Type of model**	**Description**	**Phenotypes modified**	**References**
Cellular models	Neurons with accelerated aging induced by galactose	Upregulation of antioxidant enzymes. Reduction in β amyloid protein and pro-inflammatory cytokines	[99]
Rodents	Elderly rats or rats supplemented with galactose	Decrease in oxidative stress and amyloid plaque formation. Improvement of cognitive function	[100,101]

## Abbreviations:

Aβ	Amyloid-beta
AD	Alzheimer’s disease
AGEs	Advanced glycation end-products
AKT	Protein kinase B
Ala	Alanine
ALEs	Advanced lipoxidation end-products
ARE	Antioxidant response element
ATP	Adenosine 5′-triphosphate
BBB	Blood-brain-barrier
BDNF	Brain-derived neurotrophic factor
CARNS1	Carnosine synthase 1
CARNMT	Carnosine N-methyltransferase
CN	Carnosinase
GPX	Glutathione peroxidase
CNDP	Cytosolic nonspecific dipeptidase
GSH	Glutathione
HCD	His-containing dipeptides
His	Histidine
HOCl	Hypochlorous acid
JNK	Jun N-terminal kinase
MD	Mixed dementia
MGO	Methylglyoxal
MPTP	1-methyl-4-phenyl-1,2,3,6-tetrahydropyridine
ND	Neurodegenerative disease
NMDA	N-methyl-D-aspartate
Nrf2	Nuclear factor erytroid 2-related factor 2
OS	Oxidative stress
pAD	Probable AD
PD	Parkinson’s disease
PEPT	Peptide transporter
PHT	Peptide/histidine transporter
PI3K	Phosphatidylinositol 3-kinase
POT	Proton-coupled oligopeptide transporter
ROS	Reactive oxygen species
SOD1	Superoxide dismutase 1
SLC	Solute carrier
SNpc	Substantia nigra pars compacta
6-OHDA	6-hydroxydopamine
